# P-1990. Out-of-Hospital Care Setting in a Febrile Emergency Unit of Suspected Covid 19 Patients

**DOI:** 10.1093/ofid/ofae631.2148

**Published:** 2025-01-29

**Authors:** María Cecilia Guglielmo, Valeria Aprea, Veronica Guedes, Gustavo Debaisi, Liliana Yazde Puleio, Laura Miño

**Affiliations:** Hospital de Niños Pedro de Elizalde, bs as, Buenos Aires, Argentina; Hospital de Niños Pedro de Elizalde, bs as, Buenos Aires, Argentina; Hospital de Niños Pedro de Elizalde, bs as, Buenos Aires, Argentina; Hospital de Niños Pedro de Elizalde, bs as, Buenos Aires, Argentina; Hospital de Niños Pedro de Elizalde, bs as, Buenos Aires, Argentina; Hospital de Niños Pedro de Elizalde, bs as, Buenos Aires, Argentina

## Abstract

**Background:**

In 2019, a new infection was reported in China. This coronavirus was named SARS-COV-2, causative of the 21st-century pandemic, COVID-19. Health systems adopted different strategies to cope with it.

**Objective:**

to describe the clinical-epidemiological characteristics of COVID-19 in children seen at an Emergency Febril Unit (UFU). Demographic and epidemiological characteristics of children treated at EFU of Pedro de Elizalde Children's Hospital (n = 952)

**Figure:**
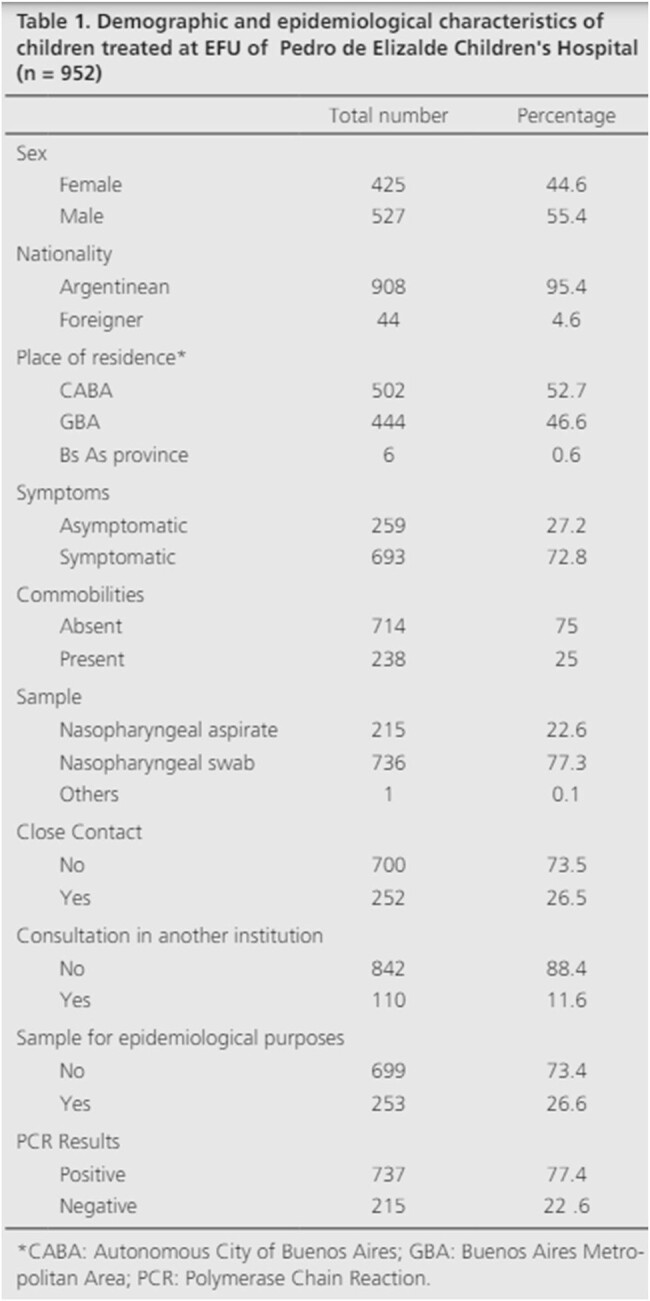

**Methods:**

Cross-sectional study in patients under 18 years of age tested for SARS-COV-2 between April 1 and June 30, 2020. All epidemiological records made at the time of consultation and the result of the Polymerase Chain Reaction (PCR) test of these patients, either by suspicion of COVID-19 or epidemiological isolation criteria, were included. Patients whose samples had been taken for SARS-COV-2 determination outside the initial time of consultation or whose epidemiological records were incomplete or did not meet the established inclusion criteria were excluded. The diagnosis of COVID-19 was made using the PCR technique for SARS-COV-2 in nasopharyngeal secretions obtained by nasopharyngeal swab or aspirate. The following variables were recorded: age, gender, place of residence, history of close contact, history of history of close contacts, travel history and comorbidities, history of institutionalization and PCR result.

Presentation of symptoms: Frequency

**Figure:**
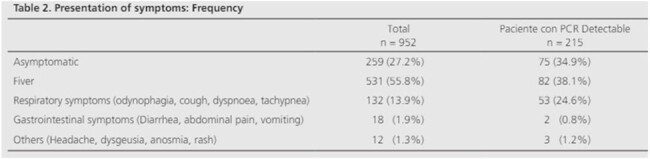

**Results:**

1,104 patients were admitted to the UFU and tested due to suspected COVID-19. 152 patients had to be excluded due to insufficient data. Of the 952 patients tested, 22.6% had a detectable result, and 71.2% of them reported close contact with confirmed cases. The mean age was 5.9 years. The 55.4% were male and 99.3% lived in the Metropolitan Area of Buenos Aires. 72.8% of the patients tested had symptoms. The time of delay in consultation was 2.17 days. 25% of the children had comorbidities.

**Conclusion:**

The availability of the UFU facilitated access and optimized the care circuit in response to demand. Children with a history of close contact and those symptomatic showed more frequently a detectable result for SARS-COV-2.

**Disclosures:**

All Authors: No reported disclosures

